# Impact of chronic kidney disease and end-stage renal disease on the mid-term adverse outcomes in diabetic patients with cardiovascular diseases

**DOI:** 10.1038/s41598-024-66655-0

**Published:** 2024-07-09

**Authors:** Chu-Lin Chou, Hui-Wen Chiu, Yung-Ho Hsu, Samuel Mon-Wei Yu, Tsan-Hon Liou, Li-Chin Sung

**Affiliations:** 1 Taipei Medical University-Research Center of Urology and Kidney (TMU-RCUK), Taipei Medical University, Taipei, Taiwan; 2 Division of Nephrology, Department of Internal Medicine, School of Medicine, College of Medicine, Taipei Medical University, Taipei, Taiwan; 3 Division of Nephrology, Department of Internal Medicine, Shuang Ho Hospital, Taipei Medical University, New Taipei City, Taiwan; 4 Division of Nephrology, Department of Internal Medicine, Hsin Kuo Min Hospital, Taipei Medical University, Taoyuan City, Taiwan; 5 Graduate Institute of Clinical Medicine, College of Medicine, Taipei Medical University, Taipei, Taiwan; 6 Department of Medical Research, Shuang Ho Hospital, Taipei Medical University, New Taipei City, Taiwan; 7 Division of Nephrology, Department of Medicine, Mount Sinai School of Medicine, New York, USA; 8 Department of Physical Medicine and Rehabilitation, Wan Fang Hospital, Taipei Medical University, Taipei, Taiwan; 9 Department of Physical Medicine and Rehabilitation, School of Medicine, College of Medicine, Taipei Medical University, Taipei, Taiwan; 10 Division of Cardiology, Department of Internal Medicine, School of Medicine, College of Medicine, Taipei Medical University, Taipei, Taiwan; 11 Division of Cardiology, Department of Internal Medicine, Shuang Ho Hospital, Taipei Medical University, New Taipei City, Taiwan; 12 Department of General Medicine, Shuang Ho Hospital, Taipei Medical University, New Taipei City, Taiwan; 13 Taipei Heart Institute, Taipei Medical University, Taipei, Taiwan

**Keywords:** Cardiovascular disease, Chronic kidney disease, Cohort study, Diabetes mellitus, End-stage renal disease, Chronic kidney disease, Cardiovascular diseases, Diabetes, Public health

## Abstract

The evidence for the impact of renal dysfunction in patients with diabetes mellitus (DM) and first cardiovascular diseases on mid-term adverse outcomes remain scarce. This study included the data of patients with DM having first atherosclerotic cardiovascular disease (ASCVD) or congestive heart failure (CHF) from the Taipei Medical University Clinical Research Database. A Cox proportional hazards regression model was used to assess the impact of chronic kidney disease (CKD) or end-stage renal disease (ESRD) on the 1-year mortality and recurrent ASCVD/CHF outcomes. We enrolled 21,320 patients with DM hospitalized for ASCVD or CHF; of them, 18,185, 2639, and 496 were assigned to the non-CKD, CKD, and ESRD groups, respectively. After propensity score matching, compared with the non-CKD group, the CKD and ESRD groups had higher mid-term all-cause mortality (adjusted hazard ratio 1.72 [95% confidence interval 1.48–1.99] and 2.77 [2.05–3.73], respectively), cardiovascular death (1.84 [1.44–2.35] and 1.87 [1.08–3.24], respectively), and recurrent hospitalization for ASCVD (1.44 [1.24–1.68] and 2.33 [1.69–3.23], respectively) and CHF (2.08 [1.75–2.47] and 1.50 [1.04–2.17], respectively). The advancing age was associated with mortality in CKD/ESRD groups. In CKD group, male sex was associated with all-cause mortality and recurrent ASCVD risk; the diuretics usage was associated with mortality and recurrent CHF risks. Our findings suggest that CKD and ESRD are significant risk factors for mid-term adverse outcomes in patients with DM and established cardiovascular diseases. Additionally, old age, male sex and diuretics usage requires attention. Further good quality studies are needed in the future.

## Introduction

Diabetes mellitus (DM) is associated with high morbidity and mortality worldwide as well as a twofold increase in cardiovascular disease (CVD)-related mortality^1^. Although DM has been considered a “coronary artery disease (CAD) risk equivalent,” this is not a universal finding, and the studies have been highly heterogeneous^2,3^.

Chronic kidney disease (CKD) is defined by the Kidney Disease Improving Global Outcomes (KDIGO) Work Group as functional or structural abnormalities of the kidneys persisting for ≥ 3 months, manifesting as decreased estimated glomerular filtration rate (eGFR; ≤ 60 mL/min/1.73 m^2^) or kidney damage (e.g., albuminuria or proteinuria)^4^. Many studies have demonstrated an exceptionally high risk of CVD in patients with CKD^5^. For patients with stage 3 CKD (eGFR: 30–59 mL/min/1.73 m^2^), the risk of cardiovascular death is ≥ tenfold higher than the risk of progression to end-stage renal disease (ESRD)^6^. The 5-year survival rate in patients with ESRD is < 40%, mainly due to CVD-related morbidity and mortality^7^. CKD is associated with CAD and an increased risk of cardiovascular complications after myocardial infarction^8,9^. Congestive heart failure (CHF) is highly prevalent among patients with CKD or ESRD. Sudden cardiac death (25%) is the leading cause of mortality in patients with ESRD and CHF^10^. Peripheral artery occlusive disease (PAOD) is prevalent among patients with CKD, affecting approximately 1 in 4 adults aged > 40 years diagnosed as having CKD^10^. Adults with PAOD are at increased risks of CVD-related hospitalization and mortality, lower-limb complications, and reduced health-related quality of life^11^. With the establishment of CKD as a powerful predictor of CVD, clinical guidelines, including those of the American Heart Association, on CVD prevention, detection, and treatment recommend considering patients with CKD as the highest-risk group^12^. As mentioned in the earlier text, patients with both DM and CKD have a considerably higher risk for CVD events.

The mechanisms underlying the strong associations between DM, CKD, and various forms of CVD seem to be complex and remain largely unknown. Moreover, the major clinical trials on CVD have typically excluded DM patients with advanced CKD or ESRD. Up to approximately 60% of patients with DM diagnosed as having CVD are likely to have coexisting renal dysfunction^12,13^. However, whether additive renal function status influences future CVD outcomes in patients with DM remains unclear. In different to the primary prevention of CVD events in patients with DM and CKD, the patients with DM and CVD are treated with healthcare provider (cardiologist), intensive lipid control, and (dual or single) anti-platelet agents. Furthermore, the management of CVD by cardiologists in patients with CKD are almost the same or partially restricted compared to patients without CKD. Therefore, in this study, we used data from the Taipei Medical University Clinical Research Database (TMUCRD) to explore the impact of CKD or ESRD status in diabetic patients with the first CVD event on mid-term adverse outcomes in Taiwan.

## Materials and methods

### Data source

The data used in this study were obtained from the institutional and clinical database of Taipei Medical University (TMU)^14^, which contains the electronic health records of more than 3 million patients from three affiliated hospitals: TMU Hospital, Wan Fang Hospital, and Shuang Ho Hospital. These hospitals have a combined capacity of 3000 beds. We collected the patients’ clinical information—including physicians’ diagnosis; prescriptions; physical and biochemical examination results; and medical expenditures of outpatient care, emergency care, and hospitalization—between January 1, 2004, and December 31, 2020. All hospitals in Taiwan are covered by the National Health Insurance program, and all medical records are reviewed by the Bureau of National Health Insurance. This rigorous regulation makes the electronic health records used in this study suitable for testing our hypothesis. This study was approved by the Institutional Review Board of Taipei Medical University (TMU-JIRB No. N202108073), and the requirement of informed consent was waived due to the de-identification of personal information in the TMURD. The study conformed to the Declaration of Helsinki. All methods were performed in accordance with the relevant guidelines and regulations. A request for the analytic methods should be sent to the corresponding author.

### Study population

**Figure 1:**
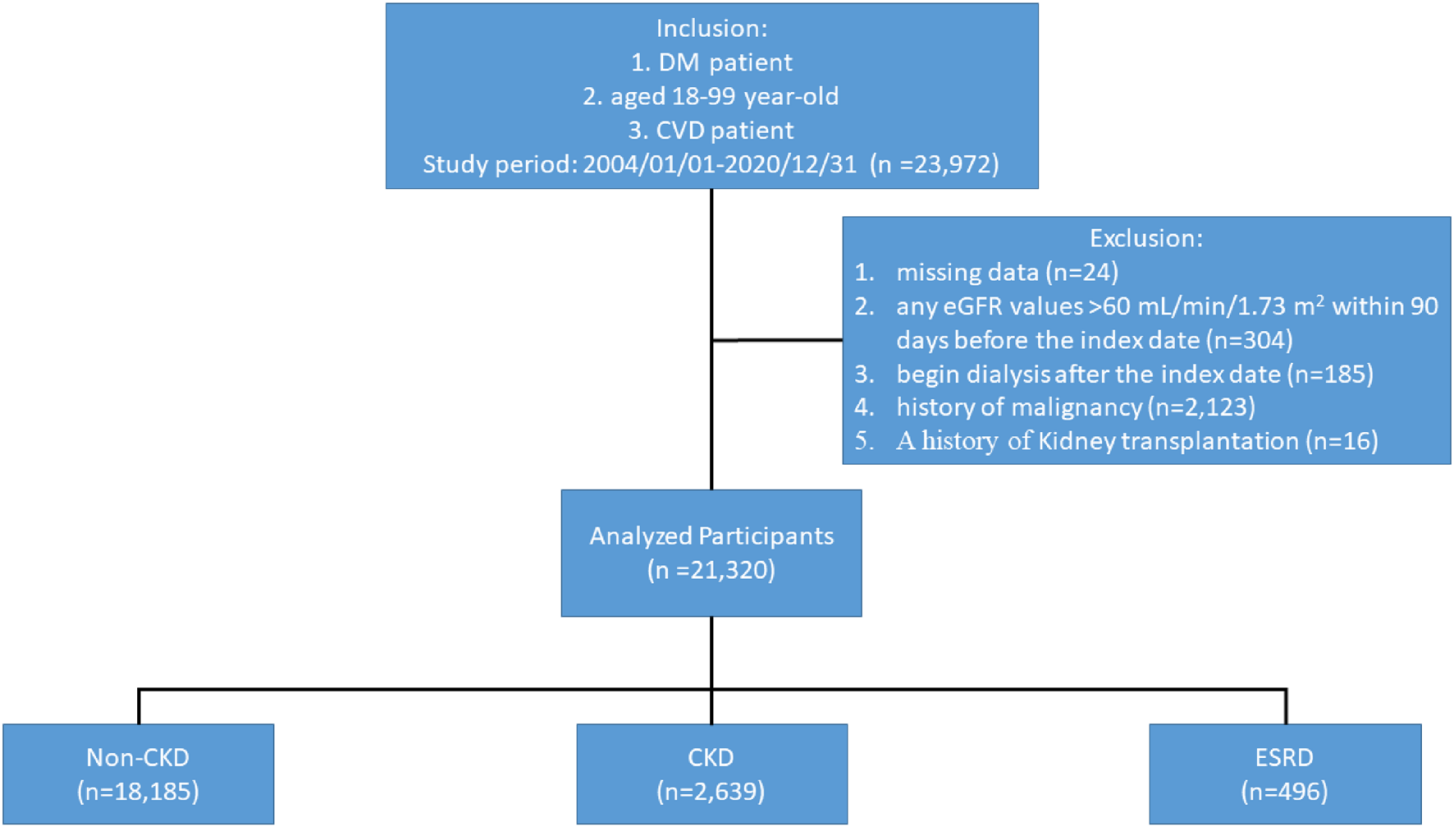
Flow chart of the patient selection process. *CKD* chronic kidney disease, *CVD* cardiovascular disease, *DM* Diabetes mellitus, *eGFR* estimated glomerular filtration rate, *ESRD* end-stage renal disease.

This study cohort included adults with type 2 DM who were aged 18–99 years and had the first clinical diagnosis of atherosclerotic CVD (ASCVD) (including stroke, CAD, and PAOD) or CHF event date (index date). The status of DM was confirmed by physician diagnosis of DM 2 times within 1 years of the index date. The CKD status was determined as an eGFR value of < 60 mL/min/1.73 m^2^ and urine protein dipstick value ≥ trace on at least 2 occasions 90 days apart before the index date. ESRD was defined as eGFR < 15 mL/min/1.73 m^2^ requiring chronic dialysis within 90 days before the index date. Next, we divided the entire cohort into non-CKD, CKD, and ESRD (i.e., under maintenance hemodialysis and peritoneal dialysis) groups (Fig. 1 and Supplementary Fig. S1). The exclusion criteria were as follows: (1) missing data for age, sex, or eGFR/urine protein, (2) any eGFR values > 60 mL/min/1.73 m^2^ within 90 days before the index date, (3) begin dialysis after the index date, (4) history of malignancy, metastatic cancer or actively treated systemic cancer, and (5) A history of kidney transplantation.

### Study endpoint

The primary outcomes were all-cause mortality, cardiovascular death, recurrent hospitalization for CHF, ASCVD events including brain stroke (hemorrhagic or ischemic stroke), acute myocardial infarction, and major adverse limb events (acute limb ischemia, major amputation, and need for surgical or peripheral revascularization). We also compared all primary outcomes in the non-CKD, CKD, and ESRD groups. All events were recorded 365 days after the index date. The follow-up period started from the index date until the occurrence of primary outcomes, loss of follow-up, 365 days after the index date, or until December 31, 2020, which-ever came first.

### Covariates

Baseline clinicodemographic characteristics within 1 year before the index date were obtained. Diagnoses were coded using *International Classification of Diseases, Ninth Revision, Clinical Modification* (ICD-9-CM) until the end of 2015 and *International Classification of Diseases, Tenth Revision, Clinical Modification* (ICD-10-CM) from 2016. Anatomical Therapeutic Chemical (ATC) codes were used to identify the medications, and medications prescribed within 1 years before the index date were recorded. Comorbidities were defined as ≥ 2 diagnostic records within 1 years before the index date. The potential risk factors for the primary outcomes included age, sex, acute kidney injury (AKI), hypertension, hyperlipidemia, atrial fibrillation (AF), chronic obstructive pulmonary disease, chronic liver disease, dementia, and medications such as angiotensin-converting enzyme inhibitors/angiotensin receptor blockers (ACEIs/ARBs, ATC: C09), beta-2 blockers (ATC: C07), calcium channel blockers (CCBs, ATC: C08), warfarin (ATC: B01AA03), rivaroxaban (ATC: B01AF01), antiplatelet drugs (ATC: B01AC), statins (ATC: C10AA, C10B), diuretics (ATC: C03, C09DA04), sulfonylureas (ATC: A10BB), metformin (ATC: A10BA), thiazolidinedione (ATC: A10BG), alpha-glucosidase inhibitors (ATC: A10BF), dipeptidyl peptidase 4 inhibitors (DPP-4is, ATC: A10BH), sodium–glucose cotransporter 2 inhibitors (SGLT-2is, ATC: A10BK), glucagon-like peptide 1 receptor agonists (GLP-1 RAs, ATC: A10BJ), and insulin (ATC: A10A) within 180 days before the index date. The AKI was defined as an abrupt decrease in kidney function over 7 days or less.

### Statistical analysis

The baseline demographic characteristics, comorbidities, medications, and the first CVD diagnosis were compared between the CKD/ESRD and non-CKD groups by using the chi-square test for categorical variables and *t* test for continuous variables. Continuous variables are presented as mean with standard deviation, whereas categorical variables are presented as counts and percentages. We conducted propensity score matching (PSM) for age, sex, comorbidities, and medication use to reduce selection bias. We matched CKD or ESRD patients to patients without CKD, using a greedy matching algorithm (without replacement) with a caliper width of 0.25 SDs of the log odds of the estimated propensity score. By calculating the standardized difference of the mean or proportion for each covariate after matching, baseline covariates with a standardized difference of less than 10% were indicative of good balance between the patients with and without diabetes. The multivariate Cox proportional hazards regression was used to estimate the crude hazard ratios (HRs), adjusted HRs (aHRs), and 95% confidence intervals (CIs) for evaluating the risks of mid-term (1-year) all-cause mortality, cardiovascular death, CHF hospitalization, and ASCVD outcomes in patients with DM after the first CVD event between the CKD/ESRD group and the non-CKD group. Subgroup analyses stratified by age, gender, BMI, HbA1c, comorbidity, and medications were performed to further examine the associations between CKD/ESRD status and all primary outcomes within these strata. Logistic regression analysis was applied to calculate propensity scores, which were used to adjust the Cox regression models to reduce bias from unmeasured confounders. Statistical analyses were performed using SAS/STAT (version 9.3; SAS Institute, Cary, NC, USA) and STATA (version 12; Stata Corp, College Station, TX, USA). *p* < 0.05 was considered statistically significant in general, and *p* < 0.001 was considered significant specifically for multiple comparisons.

## Results

### Study cohort and baseline characteristics

We identified 21,320 DM patients with newly diagnosed ASCVD or CHF and divided them into non-CKD (n = 18,185), CKD (n = 2639), and ESRD (n = 496) groups (Fig. 1), with a mean age of 64.9 ± 13.0, 73.7 ± 11.9, and 68.1 ± 12.1 years, respectively. Their baseline clinicodemographic characteristics are presented in Table 1. The percentage of men was higher than that of women in all three groups. The CKD and ESRD groups exhibited a higher prevalence of preexisting AKI, hypertension, AF, and dementia than the non-CKD group. Compared with the non-CKD group, a higher proportion of patients in the CKD and ESRD groups took ACEIs/ARBs, beta-2 blockers, CCBs, warfarin, antiplatelet drugs, diuretics, alpha-glucosidase inhibitors, DPP-4is, and insulin. Furthermore, significantly higher proportions of patients in the ESRD group took the medications, except for various oral hypoglycemic agents, than those in the CKD group. The main disease of the first CVD event in all three groups was CAD (59%–71%) (Table 1).

**Table 1:** Demographic and clinical characteristics of diabetic patients with the status of Non-CKD, CKD and ESRD before propensity score matching.

Characteristics	Overall (n = 21,320)	Non-CKD (n = 18,185)	CKD (n = 2639)	ESRD (n = 496)	*p* value^1^
Age (mean ± std)	66.1 ± 13.1	64.9 ± 13.0	73.7 ± 11.9	68.1 ± 12.1	< 0.0001
Female	10,118, 47.45%	8586, 47.21%	1286, 48.73%	246, 49.6%	0.1996
BMI	25.7 ± 5.0	25.8 ± 4.9	25.7 ± 5.3	24.5 ± 4.8	< 0.0001
Total cholesterol (mg/dL)	181.7 ± 45.2	183.6 ± 44.3	171.9 ± 49.3	168.5 ± 42.9	< 0.0001
LDL-cholesterol (mg/dL)	105.8 ± 35.7	107.2 ± 35.2	99.3 ± 37.8	90.1 ± 32.0	< 0.0001
HbA1c (%)	7.3 ± 1.8	7.3 ± 1.8	7.3 ± 1.7	7.1 ± 1.6	0.3306
Creatinine	1.4 ± 1.6	1.1 ± 0.2	2.3 ± 2.1	7.1 ± 3.8	< 0.0001
Comorbidities					
AKI	387, 1.82%	177, 0.97%	168, 6.37%	42, 8.47%	< 0.0001
Hypertension	14,483, 67.93%	12,138, 66.75%	1967, 74.54%	378, 76.21%	< 0.0001
Hyperlipidemia	10,798, 50.65%	9544, 52.48%	1108, 41.99%	146, 29.44%	< 0.0001
AF	770, 3.61%	578, 3.18%	174, 6.59%	18, 3.63%	< 0.0001
COPD	3759, 17.63%	3190, 17.54%	480, 18.19%	89, 17.94%	0.7187
CLD	2502, 11.74%	2308, 12.69%	157, 5.95%	37, 7.46%	< 0.0001
Dementia	1206, 5.66%	889, 4.89%	278, 10.53%	39, 7.86%	< 0.0001
Medications					
Diuretics	1559, 7.31%	969, 5.33%	452, 17.13%	138, 27.82%	< 0.0001
Antiplatelet	3214, 15.08%	2461, 13.53%	588, 22.28%	165, 33.27%	< 0.0001
Warfarin	159, 0.75%	110, 0.60%	35, 1.33%	14, 2.82%	< 0.0001
Rivaroxaban	73, 0.34%	37, 0.20%	34, 1.29%	2, 0.40%	< 0.0001
ACEIs/ARBs	4243, 19.90%	3371, 18.54%	707, 26.79%	165, 33.27%	< 0.0001
Beta-2 blockers	3093, 14.51%	2390, 13.14%	521, 19.74%	182, 36.69%	< 0.0001
CCBs	3509, 16.46%	2635, 14.49%	657, 24.90%	217, 43.75%	< 0.0001
Statins	2773, 13.01%	2300, 12.65%	394, 14.93%	79, 15.93%	0.0008
Metformin	3338, 15.66%	2995, 16.47%	324, 12.28%	19, 3.83%	< 0.0001
Thiazolidinedione	411, 1.93%	352, 1.94%	51, 1.93%	8, 1.61%	0.8734
Sulfonylureas	2282, 10.70%	1917, 10.54%	327, 12.39%	38, 7.66%	0.0014
AGIs	621, 2.91%	487, 2.68%	116, 4.40%	18, 3.63%	< 0.0001
DPP-4is	1344, 6.30%	898, 4.94%	376, 14.25%	70, 14.11%	< 0.0001
Insulin	2326, 10.91%	1603, 8.81%	545, 20.65%	178, 35.89%	< 0.0001
GLP-1 RAs	29, 0.14%	23, 0.13%	4, 0.15%	2, 0.40%	0.2505
SGLT-2is	0	0	0	0	
Index date CVD					
Brain stroke	2485, 11.66%	2174, 11.95%	271, 10.27%	40, 8.06%	0.0016
CAD	14,878, 69.78%	13,001, 71.49%	1586, 60.10%	291, 58.67%	< 0.0001
CHF	3302, 15.49%	2456, 13.51%	702, 26.60%	144, 29.03%	< 0.0001
PAOD	655, 3.07%	555, 3.05%	79, 2.99%	21, 4.23%	0.3154

Data are presented as mean ± standard deviation for continuous variables and number, percentage (%) for categorical variables.

^1^The chi-square or Kruskal–Wallis test (except age, which was evaluated with the Wilcoxon rank sum test).

*ACEIs* angiotensin-converting enzyme inhibitors, *AF* atrial fibrillation, *AGIs* alpha-glucosidase inhibitors, *ARBs* angiotensin receptor blockers, *AKI* acute kidney injury, *BMI* body mass index, *CAD* coronary artery disease, *CCBs* calcium channel blockers, *CHF* congestive heart failure, *CKD* chronic kidney disease, *CLD* chronic liver disease, *COPD* chronic obstructive pulmonary disease, *CVD* cardiovascular disease, *DPP-4is* dipeptidyl peptidase 4 inhibitors, *ESRD* end-stage renal disease, *GLP-1 RAs* glucagon-like peptide 1 receptor agonists, *HbA1c* glycated hemoglobin, *LDL* low density lipoprotein, *PAOD* peripheral artery occlusive disease, *SGLT-2is* sodium–glucose cotransporter 2 inhibitors.

### Group and subgroup analyses

**Figure 2:**
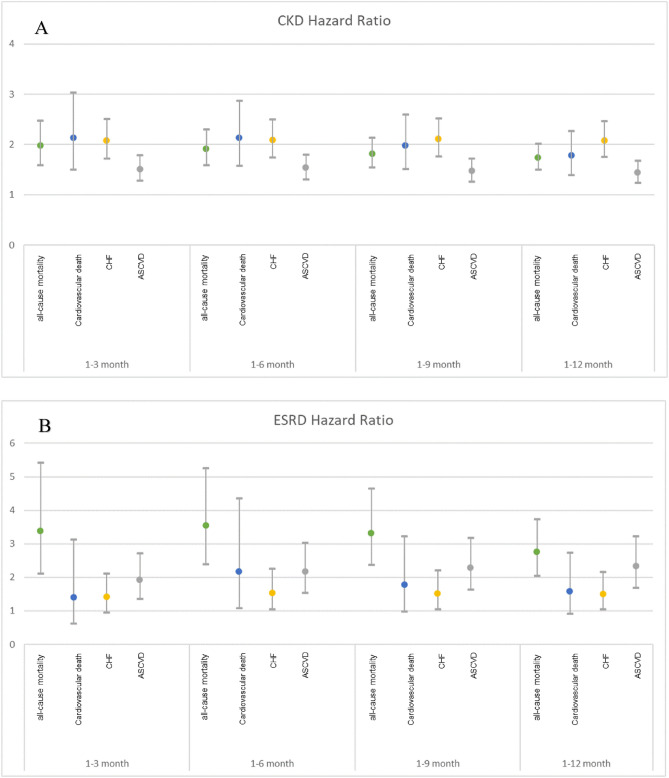
(**A**) Adjusted hazards ratios of all-cause mortality, cardiovascular death, CHF, ASCVD with 95% CI for CKD vs. non-CKD in different time intervals within 1 year. (**B**) Adjusted hazards ratios of all-cause mortality, cardiovascular death, CHF, ASCVD with 95% CI for ESRD vs. non-CKD in different time intervals within 1 year.

After PSM, no significant differences were observed in the matched variables between the non-CKD group and the CKD or ESRD group (Table 2). The aHRs for all-cause mortality, cardiovascular death, CHF hospitalization, and ASCVD outcomes in DM patients with CKD/ESRD compared with non-CKD patients in different time intervals within 1 year are presented in Fig. 2A and B. Compared with that for DM patients without CKD, the 1-year aHR for all-cause mortality was 1.72 (95% CI 1.48–1.99, *p* < 0.0001) and 2.77 (2.05–3.73, *p* < 0.0001) for DM patients with CKD and ESRD, respectively. After adjustment, compared with the non-CKD group, the CKD and ESRD groups had a higher risk of cardiovascular mortality (1-year aHR 1.84, 95% CI 1.44–2.35, *p* < 0.0001 and 1-year aHR 1.87, 95% CI 1.08–3.24, *p* = 0.0267, respectively) as well as higher 1-year recurrent hospitalization for ASCVD events (1-year aHR 1.44, 95% CI 1.24–1.68 and 1-year aHR 2.33, 95% CI 1.69–3.23, respectively, both *p* < 0.0001) and CHF (1-year aHR 2.08, 95% CI 1.75–2.47, *p* < 0.001 and 1-year aHR 1.50, 95% CI 1.04–2.17, *p* = 0.0286, respectively) (Table 3). In summary, compared with non-CKD group, greater magnitude risks of primary outcomes except CHF hospitalization were observed in the ESRD group than the CKD group.

**Table 2:** Demographic and clinical characteristics of diabetic patients with the status of Non-CKD, CKD and ESRD after 1:1 propensity score matching.

Characteristics	CKD (n = 2529)	Non-CKD (n = 2529)	SMD(%)^1^	ESRD (n = 493)	Non-CKD (n = 493)	SMD(%)^1^
Age, years	73.2 ± 11.8	73.6 ± 11.7	− 3.3	68.1 ± 12.1	69.8 ± 13.0	− 9.1
Female	1223, 48.36%	1223, 48.36%	0	245, 49.7%	245, 49.7%	0
AKI	116, 4.59%	100, 3.95%	− 3.4	41, 8.32%	35, 7.1%	− 5.8
Hypertension	1876, 74.18%	1902, 75.21%	2.3	375, 76.06%	357, 72.41%	− 8.1
Hyperlipidemia	1078, 42.63%	1064, 42.07%	− 1.1	147, 29.82%	141, 28.6%	− 2.5
AF	153, 6.05%	164, 6.48%	2.0	18, 3.65%	18, 3.65%	0
COPD	448, 17.71%	455, 17.99%	0.7	89, 18.05%	101, 20.49%	6.4
CLD	152, 6.01%	156, 6.17%	0.5	37, 7.51%	42, 8.52%	3.4
Dementia	242, 9.57%	261, 10.32%	2.8	38, 7.71%	38, 7.71%	0
Medications						
Diuretics	368, 14.55%	367, 14.51%	− 0.1	134, 27.18%	146, 29.61%	6.9
Antiplatelet	535, 21.15%	519, 20.52%	− 1.7	162, 32.86%	172, 34.89%	4.9
Warfarin	31, 1.23%	36, 1.42%	2.0	14, 2.84%	11, 2.23%	− 4.7
Rivaroxaban	19, 0.75%	18, 0.71%	− 0.4	2, 0.41%	2, 0.41%	0
ACEI/ARB	640, 25.31%	641, 25.35%	0.1	161, 32.66%	170, 34.48%	4.2
Beta-2 blocker	458, 18.11%	450, 17.79%	− 0.9	178, 36.11%	184, 37.32%	2.9
CCB	577, 22.82%	575, 22.74%	− 0.2	213, 43.2%	219, 44.42%	2.8
Statin	362, 14.31%	362, 14.31%	0	77, 15.62%	80, 16.23%	1.7
Metformin	317, 12.53%	336, 13.29%	2.1	19, 3.85%	25, 5.07%	4.1
Thiazolidinedione	49, 1.94%	44, 1.74%	− 1.4	8, 1.62%	12, 2.43%	6.2
Sulfonylureas	301, 11.9%	307, 12.14%	0.7	37, 7.51%	30, 6.09%	− 4.9
AGIs	100, 3.95%	99, 3.91%	− 0.2	18, 3.65%	22, 4.46%	4.6
DPP4is	290, 11.47%	259, 10.24%	− 4.2	66, 13.39%	59, 11.97%	− 4.9
Insulin	461, 18.23%	470, 18.58%	1.0	174, 35.29%	186, 37.73%	6.2
GLP-1 RAs	2, 0.08%	0, 0%	− 2.2	2, 0.41%	2, 0.41%	0
Index date CVD						
Brain stroke	263, 10.35%	296, 11.65%	4.1	40, 1.57%	40, 1.57%	0
CAD	1558, 61.34%	1539, 60.59%	− 1.6	289, 11.37%	288, 11.33%	− 0.4
CHF	632, 24.88%	627, 24.69%	− 0.5	144, 5.67%	142, 5.59%	− 1.0
PAOD	76, 2.99%	67, 2.64%	− 2.1	20, 0.79%	23, 0.91%	3.3

Data are presented as mean ± standard deviation for continuous variables and number, percentage (%) for categorical variables.

^1^Standardized mean difference.

*ACEIs* angiotensin-converting enzyme inhibitors, *AF* atrial fibrillation, *AGIs* alpha-glucosidase inhibitors, *ARBs* angiotensin receptor blockers, *AKI* acute kidney injury, *CAD* coronary artery disease, *CCBs* calcium channel blockers, *CHF* congestive heart failure, *CKD* chronic kidney disease, *CLD* chronic liver disease, *COPD* chronic obstructive pulmonary disease, *CVD* cardiovascular disease, *DPP-4is* dipeptidyl peptidase 4 inhibitors, *ESRD* end-stage renal disease, *GLP-1 RAs* glucagon-like peptide 1 receptor agonists, *PAOD* peripheral artery occlusive disease, *SGLT-2is* sodium–glucose cotransporter 2 inhibitors.

**Table 3:** The adjusted hazards ratios of all-cause mortality, cardiovascular death, CHF, ASCVD with 95% CI for the comparison of CKD vs. Non-CKD and ESRD vs. Non-CKD groups in one year.

Adverse outcomes	CKD (n = 2529) vs. Non-CKD (N = 2529)				ESRD (n = 493) vs. Non-CKD (n = 493)			
	CKD (n, %)	Non-CKD (n, %)	Adjusted HR^†^ (95% CI)	*P* value	ESRD (n, %)	Non-CKD (n, %)	Adjusted HR^†^ (95% CI)	*P* value
All-cause mortality	465, 18.39%	295, 11.66%	1.72 (1.48, 1.99)	< 0.0001	140, 28.40%	69, 14%	2.77 (2.05, 3.73)	< 0.0001
Cardiovascular death	179, 7.08%	103, 4.07%	1.84 (1.44, 2.35)	< 0.0001	34, 6.9%	23, 4.67%	1.87 (1.08, 3.24)	0.0267
CHF	385, 15.22%	198, 7.83%	2.08 (1.75, 2.47)	< 0.0001	71, 14.4%	52, 10.55%	1.50 (1.04, 2.17)	0.0286
ASCVD^1^	400, 15.82%	287, 11.35%	1.44 (1.24, 1.68)	< 0.0001	113, 22.92%	56, 11.36%	2.33 (1.69, 3.23)	< 0.0001

*ASCVD* atherosclerotic cardiovascular disease, *CHF* congestive heart failure, *CKD* chronic kidney disease, *ESRD* end-stage renal disease, *HR* hazard ratio.

^†^Main model was adjusted for all covariates (listed in Table 2) such as gender; age; Comorbidity (AKI; Hypertension; Hyperlipidemia; AF; COPD; CLD; Dementia); Medications (Diuretics; Antiplatelet; Warfarin; Rivaroxaban; ACEI/ARB; Beta-2 blocker; CCB; Statin; Metformin; Thiazolidinedione; Sulfonylureas; AGIs; DPP4is; Insulin; GLP-1 RAs); Index date CVD (brain stroke; CAD; CHF; PAOD).

^1^The study events for ASCVD: (1) brain stroke (hemorrhagic stroke and ischemic stroke), (2) acute myocardial infarction, and (3) major adverse limb events (acute limb ischemia, major amputation, need for surgical or peripheral revascularization of peripheral artery occlusive disease).

*ACEIs* angiotensin-converting enzyme inhibitors, *AF* atrial fibrillation, *AGIs* alpha-glucosidase inhibitors, *ARB* angiotensin receptor blockers, *AKI* acute kidney injury, *ASCVD* atherosclerotic cardiovascular disease, *CAD* coronary artery disease, *CCBs* calcium channel blockers, *CHF* congestive heart failure, *CKD* chronic kidney disease, *CLD* chronic liver disease, *COPD* chronic obstructive pulmonary disease, *Cr* creatine, *DPP-4is* dipeptidyl peptidase 4 inhibitors, *eGFR* estimated Glomerular filtration rate, *GLP-1 RAs* glucagon-like peptide 1 receptor agonists, *LDL* low-density lipoprotein, *HbA1c* glycated hemoglobin, *PAOD* peripheral artery occlusive disease.

Furthermore, Supplementary Tables 1 and 2 display the results of the subgroup analysis, which was conducted through Cox proportional hazards regression, of each primary end point. Advancing age and male sex were independently associated with mortality and certain adverse CVD outcomes in CKD group. The advancing age was associated with mortality in ESRD group. The preexisting AKI and AF were associated with higher CHF risk in CKD group than non-CKD group. No medication was noted to be beneficial for preventing all primary outcomes. The need of diuretics usage or inappropriate use of diuretics were associated with mortality and CHF risks in CKD group. Furthermore, certain traditional risk factors, such as hypertension and hyperlipidemia, had an inverse association with mortality in patients with DM.

## Discussion

In this retrospective cohort study, we explored the potential detrimental and predictive effects of renal dysfunction in patients with DM. First, DM patients with CKD or ESRD have higher risks of mid-term all-cause mortality, cardiovascular death, CHF hospitalization, and ASCVD outcomes compared with those without CKD after multivariate adjustment. Second, the ESRD group exhibited higher risks of all-cause mortality, cardiovascular death, and ASCVD outcomes than the CKD group, but the ESRD group had less risk of CHF. Third, advancing age was associated with mortality in diabetic patients with CKD or ESRD. The male sex was associated with all-cause mortality and ASCVD outcomes in CKD group. The need of diuretics usage or inappropriate use of diuretics were associated with mortality and CHF risks in CKD group. Our findings support the evidence of renal dysfunction impact the outcomes in patients with DM and established CVD.

CVD is a major cause of mortality and morbidity among people with DM. The macrovascular complications of DM mainly include ASCVD, typically manifesting as CAD, PAOD, and stroke^15^. Type 2 DM is a significant predictor of CHF, independent of the simultaneous presence of hypertension and CAD. DM induces CHF through various mechanisms, including CAD, microvascular complications, arterial thickening, endothelial, and vasomotor dysfunction. Compared with people without DM, CHF risk is 2–4 times higher in men with DM and 5 times higher in women with DM^15–17^. In summary, atherosclerotic CVD and CHF, is present in 32.2% of patients with type 2 DM^18^.

DM is the most well-known cause of CKD and ESRD, and > 50% of people with DM are likely to develop CKD^19^. CKD is associated with significant mortality risk in patients with DM, and the risk of cardiovascular death increases as eGFR declines^19^. CHF incidence is threefold higher in patients with eGFR < 60 mL/min/1.7 m^2^^20^. In patients with DM, the high risks of all-cause mortality and adverse CVD outcomes are mainly due to CKD and related complications^7,21–24^. A meta-analysis concluded that all CKD stages were associated with a 43% higher risk of incident stroke^22^. In another study, 73% of patients on hemodialysis had developed significant CAD^23^. A study revealed cumulative increases in atherosclerosis and thrombosis risks during the transition from CKD to ESRD^25^.

In this study, we explored whether CKD and ESRD increase the risks of all-cause mortality and future CVD events in diabetic patients with established CVD. A cohort study including 208,792 patients with DM discovered that heart disease (CAD and CHF), stroke, and moderate CKD (eGFR = 30–59 mL/min/1.73 m^2^), and their combination had independent and cumulative effects on all-cause mortality over a median follow-up period of 8.5 years^26^. The mortality risks increased with the increase in the number of the aforementioned conditions^26^. A similar trend of greater magnitude was observed for severe CKD (eGFR < 30 mL/min/1.73 m^2^). These results are consistent with our data of all-cause mortality, cardiovascular death and ASCVD. However, the risk of CHF hospitalization is not consistent with the trend (ESRD > CKD), which is due to the beneficial effect of regular fluid removal in patients with chronic dialysis. AKI increased hospitalization for CHF, not ASCVD, risk in our diabetic patients with CKD, this is compatible with previous cohort study^27^.

The mechanisms for the impact of CKD and ESRD on the various forms of CVD outcomes are not fully understood. Traditional risk factors for CVD, although also prevalent among those with DM and CKD, do not fully account for the increased mortality and CVD events. Furthermore, certain traditional risk factors such as hypertension, hyperlipidemia, and obesity have actually been observed to be inversely associated with CVD outcomes in patients with CKD^28^, which is consistent with our results. Theoretically, the incidence of HTN and hyperlipidemia in CKD or ESRD increased as renal function worsen. This paradox may be explained by anemia, systemic inflammation and/or malnutrition, which are associated with lower cholesterol/low blood pressure levels and higher mortality^29,30^. AF increased risk of CHF in the CKD group and cardiovascular death in the ESRD group. In previous reports, the AF increase CHF risk with a subdistribution hazard ratio 2.64 in patients with a decreased eGFR < 90 mL/min/1.73 m^2^^31^. Besides, AF is associated with greater all-cause and cardiovascular mortality in one cohort study of long-term hemodialysis patients^32^. The comorbidities of chronic obstructive pulmonary disease (COPD) or dementia increased ASCVD risks in our CKD group patients, which are partially compatible with previous cohort studies^33,34^. These diseases share common vascular and metabolic milieu that consists of chronic inflammation, oxidative stress, and endothelial dysfunction. The early identification of those with COPD or dementia who present with CVD events is crucial, as subsequent tailoring of treatment strategies can potentially improve outcomes. However, the phenomenon was not seen in ESRD group, which may be due to higher vascular inflammation/calcification risks in patients with ESRD than COPD or dementia per se. One meta-analysis including over 1.2 million patients revealed a linear association between lower eGFR and the risk of cardiovascular mortality^19^. The American Diabetes Association (ADA)’s current clinical practice guidelines recommend the evaluation and management of potential CKD complications, including elevated blood pressure, volume overload, electrolyte abnormalities, metabolic acidosis, anemia, and metabolic bone disease, when eGFR is < 60 mL/min/1.73 m^35^. Other risk factors for CVD in patients with CKD include disturbance of calcium–phosphate homeostasis, anemia, arterial calcification, hyperaldosteronism, chronic inflammation, abnormal nitric oxide metabolism, and endothelial dysfunction^5,10,35,36^. Moreover, both DM and CKD have hypercoagulable conditions and are associated with high bleeding risk simultaneously^10,37^, which may partially explain the mechanisms.

The mainstay of treatment for DM plus CKD is the blockade of the renin–angiotensin–aldosterone system and control of hypertension, hyperglycemia, and atherogenic dyslipidemia^10^. The treatment of choice for CVD depends on its etiology and severity. More than two-thirds of mortality from CKD is a result of sudden cardiac death, which is mainly caused by ventricular arrhythmia^5^. Prevention of rapid volume change, treatment of electrolyte disturbance, and treatment with beta-2 blockers may be beneficial for these patients^5^, which is compatible with our data for the detrimental effect of diuretics and beneficial effect of beta-2 blockers. Furthermore, diuretic use is associated with renin-angiotensin–aldosterone/sympathetic system activation, insufficient plasma volume and increased blood viscosity, which is harmful for diabetic patients with CVD and CKD. Among the anti-DM medications, metformin, SGLT-2is, and the GLP-1 RA liraglutide have demonstrated survival benefits^38^. Metformin use was associated with lower rates of all-cause mortality in a cohort of 12,156 patients with DM^39^. Most guidelines suggest initial treatment with metformin, whereas the European Society of Cardiology guidelines recommend initial therapy with an SGLT-2i for patients with high CVD risk^40,41^. The ADA recommends SGLT-2is or GLP-1 RAs for patients with DM who have ASCVD, CKD, or CHF^41^. The current KDIGO guideline recommends using both metformin and an SGLT-2is for most patients with DM and CKD with an eGFR ≥ 30 mL/min/1.73 m^41^. However, metformin and SGLT-2is are absolutely contraindicated in patients with severe CKD (eGFR < 30 mL/min/1.73 m^2^), which limits their clinical application. At present, statins are first-line agents for patients with ASCVD for optionally achieving serum low-density lipoprotein cholesterol (LDL-C) < 70 mg/dL. However, whether statins should be discontinued once dialysis commences remains unclear. PCSK9 inhibitors should be used as a second-line therapy in patients at very high risk of not achieving the optimal LDL-C level after treatment with a maximum tolerated dose of a statin and ezetimibe^42^. We did not find any beneficial/detrimental effects of antiplatelet agents in our study population. However, previous report showed that the use of antiplatelet agents probably reduced myocardial infarction and increased major bleeding, but do not appear to reduce all-cause and cardiovascular mortality in patients with CKD or ESRD^43^. Hypertension, CHF and ASCVD should preferably be managed with ACEIs or ARBs in diabetic patients with CKD^5,7,15,38,44^. Clinical guidelines have long recommended a target blood pressure of < 130/80 mmHg for patients with DM^10^. The 2022 Taiwan Society of Cardiology hypertension guidelines and the KDIGO 2021 clinical practice guidelines recommend a target blood pressure of < 120/80 mmHg, if tolerable^45,46^. In the present study, the ACEIs/ARBs usage was low compared with the prevalence of HTN in the CKD (25.31% vs 74.18%) and ESRD (32.66% vs 72.06%) groups, respectively (Table 2). In a large multicenter cohort, 57% of the study population with advanced CKD (eGFRs < 30 mL/min/1.73 m^2^) were consistently using ACEIs/ARBs^47^. Besides, in patients with predialysis advanced CKD, the ACEIs/ARBs usage was lower in patients with comorbidity of ASCVD (CAD 26.1% and stroke 19.2%) than in patients with DM (57.7%)^44^. Therefore, the risk factor modifications and medical treatments were relative conservative in our diabetic patients with CVD and CKD, leading to a certain degree of residual cardiovascular risks. Further understanding the pathogenesis and comprehensive CKD/CVD case management is critical to improving outcomes.

The strengths of our study are the large sample size recruited from the TMUCRD, adjustment for primary risk factors, and subgroup analyses. In addition, we minimized selection bias and the effects of confounders by PSM for age, sex, comorbidities, medications, and index date CVD.

This study also has some limitations. First, the TMUCRD does not include data on lifestyle, medication nonadherence, and personal habits. Second, given that this study was a real-world observational study, bias attributable to unmeasured confounders may be present. However, this study design where a randomized controlled trial is unsuitable, such as heterogeneity in comorbidity, prescription drug selections, and limitations, a genetic disposition to CVD/CKD/ESRD, and fluctuation between different stages of CKD over time. In this study, we only aimed to demonstrate a causal relationship between renal dysfunction and future adverse outcomes. Third, our results may be generalizable only to Taiwan and other countries with universal health insurance coverage.

## Conclusions

Our results revealed that in patients with DM having ASCVD or CHF, the presence of CKD and ESRD carries an additive risk that can determine the mid-term mortality and future adverse cardiovascular outcomes. Additionally, old age, male sex and diuretics usage requires attention and evidence-based management to improve clinical outcomes. Understanding the comorbid conditions with DM, namely CVD and CKD, is critical for clinical decision-making, pharmacologic treatment strategies, and team-based work.

### Supplementary Information

Supplementary Information.

## Data Availability

Data are available from the Taipei Medical University Clinical Research Database, published by Taipei Medical University (TMU). Because of the legal restrictions imposed by the government of Taiwan in relation to the Personal Information Protection Act, data cannot be made publicly available. The datasets collected and analyzed in our study are available from the corresponding author (Li-Chin Sung, email: 10204@s.tmu.edu.tw) on reasonable request.

## References

[1] Rao Kondapally Seshasai S (2011). Diabetes mellitus, fasting glucose, and risk of cause-specific death. N. Engl. J. Med..

[2] Haffner SM, Lehto S, Rönnemaa T, Pyörälä K, Laakso M (1998). Mortality from coronary heart disease in subjects with type 2 diabetes and in nondiabetic subjects with and without prior myocardial infarction. N. Engl. J. Med..

[3] Lee CD, Folsom AR, Pankow JS, Brancati FL (2004). Cardiovascular events in diabetic and nondiabetic adults with or without history of myocardial infarction. Circulation.

[4] Levey AS (2005). Definition and classification of chronic kidney disease: A position statement from kidney disease: Improving global outcomes (KDIGO). Kidney Int..

[5] Jankowski J, Floege J, Fliser D, Bohm M, Marx N (2021). Cardiovascular disease in chronic kidney disease: Pathophysiological insights and therapeutic options. Circulation.

[6] Eriksen BO, Ingebretsen OC (2006). The progression of chronic kidney disease: A 10-year population-based study of the effects of gender and age. Kidney Int..

[7] Maqbool M, Cooper ME, Jandeleit-Dahm KAM (2018). Cardiovascular disease and diabetic kidney disease. Semin. Nephrol..

[8] Chonchol M (2008). Chronic kidney disease is associated with angiographic coronary artery disease. Am. J. Nephrol..

[9] Anavekar NS (2004). Relation between renal dysfunction and cardiovascular outcomes after myocardial infarction. N. Engl. J. Med..

[10] Palsson R, Patel UD (2014). Cardiovascular complications of diabetic kidney disease. Adv. Chronic Kidney Dis..

[11] Bourrier M (2020). Peripheral artery disease: Its adverse consequences with and without CKD. Am. J. Kidney Dis..

[12] Sarnak MJ (2003). Kidney disease as a risk factor for development of cardiovascular disease: A statement from the American Heart Association Councils on Kidney in Cardiovascular Disease, High Blood Pressure Research, Clinical Cardiology, and Epidemiology and Prevention. Circulation.

[13] McClellan WM, Langston RD, Presley R (2004). Medicare patients with cardiovascular disease have a high prevalence of chronic kidney disease and a high rate of progression to end-stage renal disease. J. Am. Soc. Nephrol. JASN.

[14] Lin YC (2019). Effect of weight loss on the estimated glomerular filtration rates of obese patients at risk of chronic kidney disease: The RIGOR-TMU study. J. Cachexia Sarcop. Muscle.

[15] LowWang CC, Hess CN, Hiatt WR, Goldfine AB (2016). Clinical update: Cardiovascular disease in diabetes mellitus: Atherosclerotic cardiovascular disease and heart failure in type 2 diabetes mellitus—mechanisms, management, and clinical considerations. Circulation.

[16] Seferović PM (2018). Type 2 diabetes mellitus and heart failure: A position statement from the Heart Failure Association of the European Society of Cardiology. Eur. J. Heart Fail..

[17] Kannel WB, McGee DL (1979). Diabetes and cardiovascular disease: The Framingham study. JAMA.

[18] Einarson TR, Acs A, Ludwig C, Panton UH (2018). Prevalence of cardiovascular disease in type 2 diabetes: A systematic literature review of scientific evidence from across the world in 2007–2017. Cardiovasc. Diabetol..

[19] Matsushita K (2010). Association of estimated glomerular filtration rate and albuminuria with all-cause and cardiovascular mortality in general population cohorts: A collaborative meta-analysis. Lancet.

[20] Kottgen A (2007). Reduced kidney function as a risk factor for incident heart failure: The atherosclerosis risk in communities (ARIC) study. J. Am. Soc. Nephrol. JASN.

[21] Schiffrin EL, Lipman ML, Mann JF (2007). Chronic kidney disease: Effects on the cardiovascular system. Circulation.

[22] Lee M (2010). Low glomerular filtration rate and risk of stroke: Meta-analysis. BMJ.

[23] Rostand SG, Kirk KA, Rutsky EA (1984). Dialysis-associated ischemic heart disease: Insights from coronary angiography. Kidney Int..

[24] Wang HH, Hung SY, Sung JM, Hung KY, Wang JD (2014). Risk of stroke in long-term dialysis patients compared with the general population. Am. J. Kidney Dis..

[25] Saran R (2016). US renal data system 2015 annual data report: Epidemiology of kidney disease in the United States. Am. J. Kidney Dis..

[26] Wan EYF (2020). The impact of cardiovascular disease and chronic kidney disease on life expectancy and direct medical cost in a 10-year diabetes cohort study. Diabetes Care.

[27] Ikizler TA (2021). A prospective cohort study of acute kidney injury and kidney outcomes, cardiovascular events, and death. Kidney Int..

[28] Kovesdy CP, Anderson JE (2007). Reverse epidemiology in patients with chronic kidney disease who are not yet on dialysis. Semin. Dial..

[29] Liu Y (2004). Association between cholesterol level and mortality in dialysis patients: Role of inflammation and malnutrition. JAMA.

[30] Kovesdy CP, Trivedi BK, Kalantar-Zadeh K, Anderson JE (2006). Association of low blood pressure with increased mortality in patients with moderate to severe chronic kidney disease. Nephrol. Dial. Transplant..

[31] Massicotte-Azarniouch D (2018). Incident atrial fibrillation and the risk of congestive heart failure, myocardial infarction, end-stage kidney disease, and mortality among patients with a decreased estimated GFR. Am. J. Kidney Dis..

[32] Genovesi S (2008). Atrial fibrillation and morbidity and mortality in a cohort of long-term hemodialysis patients. Am. J. Kidney Dis..

[33] Trudzinski FC (2019). Consequences of chronic kidney disease in chronic obstructive pulmonary disease. Respir. Res..

[34] Kodesh A (2023). The independent impact of dementia in patients undergoing percutaneous coronary intervention for acute myocardial infarction. Clin. Cardiol..

[35] American Diabetes Association (2018). Microvascular complications and foot care: Standards of medical care in diabetes-2018. Diabetes Care.

[36] Hruska KA, Sugatani T, Agapova O, Fang Y (2017). The chronic kidney disease: Mineral bone disorder (CKD-MBD): Advances in pathophysiology. Bone.

[37] Jalal DI, Chonchol M, Targher G (2010). Disorders of hemostasis associated with chronic kidney disease. Semin. Thromb. Hemost..

[38] Anders HJ, Huber TB, Isermann B, Schiffer M (2018). CKD in diabetes: Diabetic kidney disease versus nondiabetic kidney disease. Nat. Rev. Nephrol..

[39] Bergmark BA (2019). Metformin use and clinical outcomes among patients with diabetes mellitus with or without heart failure or kidney dysfunction: Observations from the SAVOR-TIMI 53 trial. Circulation.

[40] Cosentino F (2020). 2019 ESC guidelines on diabetes, pre-diabetes, and cardiovascular diseases developed in collaboration with the EASD. Eur. Heart J..

[41] Kidney Disease: Improving Global Outcomes Diabetes Work Group (2020). KDIGO 2020 clinical practice guideline for diabetes management in chronic kidney disease. Kidney Int..

[42] Mach F (2020). 2019 ESC/EAS guidelines for the management of dyslipidaemias: Lipid modification to reduce cardiovascular risk. Eur. Heart J..

[43] Natale P (2022). Antiplatelet agents for chronic kidney disease. Cochrane Database Syst. Rev..

[44] Hsu TW (2014). Renoprotective effect of renin-angiotensin-aldosterone system blockade in patients with predialysis advanced chronic kidney disease, hypertension, and anemia. JAMA Intern. Med..

[45] Wang TD (2022). 2022 guidelines of the Taiwan society of cardiology and the Taiwan hypertension society for the management of hypertension. Acta Cardiol. Sin..

[46] Cheung AK (2021). Executive summary of the KDIGO 2021 clinical practice guideline for the management of blood pressure in chronic kidney disease. Kidney Int..

[47] Arora N, Katz R, Bansal N (2020). ACE inhibitor/angiotensin receptor blocker use patterns in advanced CKD and risk of kidney failure and death. Kidney Med..

