# Supplementary material for: Impact of chronic kidney disease and end-stage renal disease on the mid-term adverse outcomes in diabetic patients with cardiovascular diseases
Source: Sci Rep. 2024 Jul 9;14:15770. doi: 10.1038/s41598-024-66655-0 (PMC11233494; doi:10.1038/s41598-024-66655-0)
Supplement: Supplementary file 1 — Supplementary Information. [file 41598_2024_66655_MOESM1_ESM.pdf]

Impact of chronic kidney disease and end-stage renal disease on the mid-term adverse outcomes in diabetic patients with cardiovascular diseases

Chu-Lin Chou, Hui-Wen Chiu, Yung-Ho Hsu, Samuel Mon-Wei Yu, Tsan-Hon Liou, Li-Chin Sung*

***Corresponding:**
Li-Chin Sung, MD, PhD (10204@s.tmu.edu.tw)

Supplementary Information:

Supplementary Figure S1

Supplementary Table S1

Supplementary Table S2

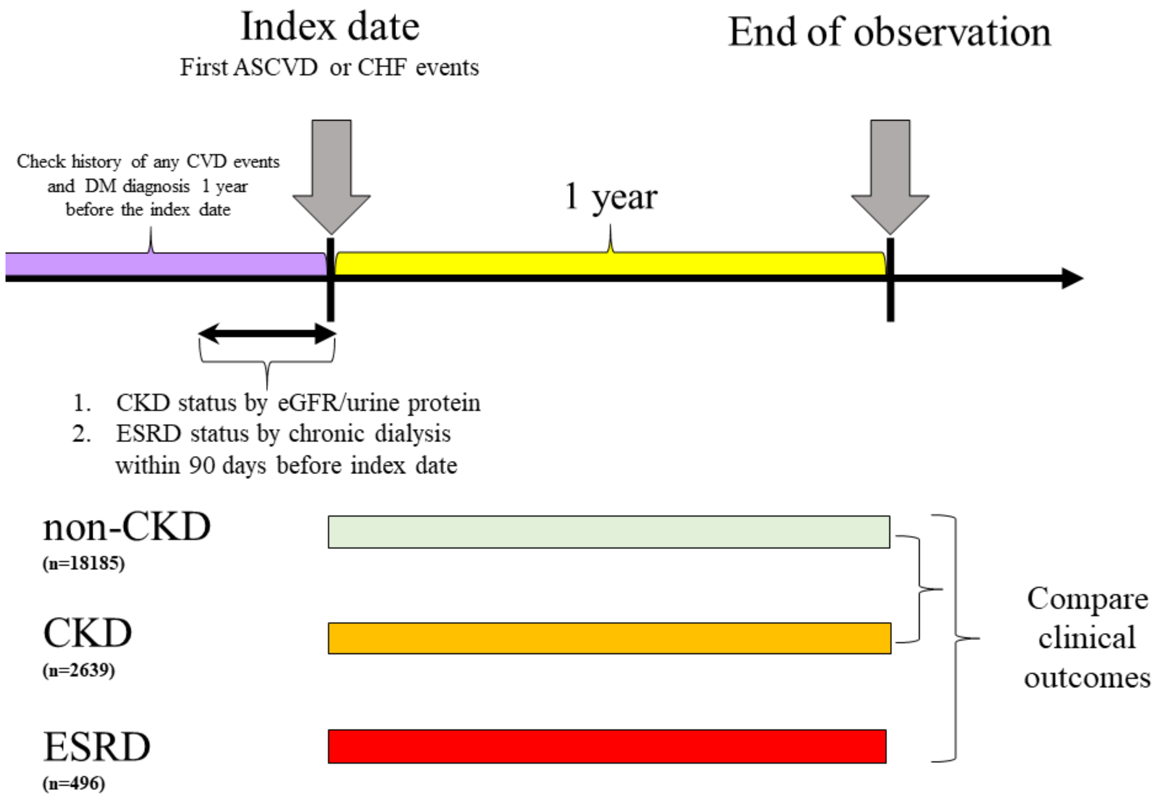

Supplementary Figure S1. The diagram for the relationship of index date, the determination of non-CKD, CKD or ESRD status, clinical outcomes comparison and follow-up period in DM patients with first ASCVD or CHF events.

Supplementary Table 1. Subgroup analysis of adjusted hazard ratio in 1-year adverse outcomes for the comparison of CKD and non-CKD groups

		Adverse outcomes			
Variable	reference	all-cause mortality	cardiovascular death	Hospitalization for CHF	[1]Hospitalization for ASCVD
CKD	Non-CKD	1.72*** (1.48, 1.99)	1.84*** (1.44, 2.35)	2.08*** (1.75, 2.47)	1.44*** (1.24, 1.68)
Age (year)		1.05*** (1.04, 1.05)	1.04*** (1.03, 1.05)	1.01* (1.00, 1.02)	1.00 (1.00, 1.01)
Male	Female	1.20* (1.04, 1.39)	1.06 (0.83, 1.34)	0.95 (0.81, 1.12)	1.49*** (1.27, 1.74)
Comorbidity					
AKI	normal	1.33 (0.98, 1.80)	0.83 (0.44, 1.56)	1.61** (1.17, 2.21)	1.10 (0.76, 1.58)
Hypertension	normal	0.59*** (0.50, 0.69)	0.55*** (0.43, 0.71)	0.96 (0.79, 1.17)	0.86 (0.72, 1.02)
Hyperlipidemia	normal	0.55*** (0.46, 0.66)	0.66** (0.50, 0.87)	0.79** (0.66, 0.94)	0.93 (0.79, 1.09)
AF	normal	0.92 (0.69, 1.22)	1.01 (0.63, 1.60)	2.35*** (1.84, 3.01)	1.19 (0.89, 1.59)
COPD	normal	0.94 (0.78, 1.12)	0.72 (0.52, 1.01)	1.12 (0.91, 1.38)	0.67** (0.53, 0.84)
CLD	normal	0.87 (0.60, 1.25)	0.70 (0.36, 1.36)	0.95 (0.66, 1.37)	0.71 (0.49, 1.03)
Dementia	normal	1.23 (1.00, 1.51)	1.25 (0.88, 1.76)	0.88 (0.67, 1.16)	1.35* (1.06, 1.71)
Medications					
Diuretics	Not use	1.42** (1.15, 1.77)	1.67** (1.14, 2.46)	1.56** (1.22, 2.01)	1.21 (0.93, 1.56)
Antiplatelet	Not use	0.96 (0.78, 1.18)	0.92 (0.63, 1.34)	0.90 (0.70, 1.14)	1.14 (0.90, 1.44)
Warfarin	Not use	0.83 (0.47, 1.49)	0.85 (0.31, 2.34)	0.37* (0.15, 0.90)	0.74 (0.37, 1.52)
Rivaroxaban	Not use	1.14 (0.58, 2.23)	0.86 (0.21, 3.54)	0.71 (0.29, 1.75)	1.52 (0.71, 3.28)
ACEIs/ARBs	Not use	0.89 (0.71, 1.11)	0.74 (0.50, 1.10)	1.01 (0.79, 1.30)	0.96 (0.75, 1.23)
Beta-2 blockers	Not use	0.94 (0.75, 1.18)	0.78 (0.51, 1.19)	0.85 (0.65, 1.10)	0.69** (0.53, 0.90)
CCBs	Not use	1.21 (0.98, 1.49)	1.18 (0.81, 1.72)	1.06 (0.84, 1.36)	0.97 (0.77, 1.24)
Statins	Not use	0.78 (0.59, 1.01)	0.62 (0.37, 1.04)	0.90 (0.68, 1.20)	1.13 (0.87, 1.48)
Metformin	Not use	0.97 (0.76, 1.24)	0.93 (0.59, 1.46)	1.17 (0.89, 1.54)	0.89 (0.67, 1.17)
Thiazolidinedione	Not use	0.93 (0.54, 1.57)	0.97 (0.38, 2.45)	0.38* (0.16, 0.93)	0.70 (0.37, 1.34)
Sulfonylureas	Not use	1.11 (0.87, 1.41)	1.00 (0.64, 1.56)	0.99 (0.74, 1.31)	1.14 (0.86, 1.50)
AGIs	Not use	0.96 (0.68, 1.38)	1.40 (0.78, 2.51)	1.51* (1.03, 2.21)	1.18 (0.79, 1.75)
DPP-4is	Not use	1.15 (0.89, 1.48)	1.20 (0.75, 1.91)	1.03 (0.77, 1.38)	1.00 (0.75, 1.34)
Insulin	Not use	1.51** (1.22, 1.88)	1.17 (0.79, 1.72)	1.11 (0.86, 1.42)	0.99 (0.77, 1.27)
GLP-1 RAs	Not use	3.99 (0.51, 31.25)	12.9* (1.35, 123.23)		

Cox proportional hazards regression, * $p < 0.05$ ** $p < 0.01$ *** $p < 0.0001$

[1]The study events for ASCVD:

(1) brain stroke (hemorrhagic stroke and ischemic stroke),

(2) acute myocardial infarction, and

(3) major adverse limb events (acute limb ischemia, major amputation, need for surgical or peripheral revascularization of peripheral artery occlusive disease).

1:1 Matching on propensity score with index date, age, sex, comorbidities, and medications.

Abbreviations: ACEIs, angiotensin-converting enzyme inhibitors; AF, atrial fibrillation; AGIs, alpha-glucosidase inhibitors; ARB, angiotensin receptor blockers; AKI, acute kidney injury; ASCVD, atherosclerotic cardiovascular disease; CCBs, calcium channel blockers; CHF, congestive heart failure; CKD, chronic kidney disease; CLD, chronic liver disease; COPD, chronic obstructive pulmonary disease; DPP-4is, dipeptidyl peptidase 4 inhibitors; GLP-1 RAs, glucagon-like peptide 1 receptor agonists

Supplementary Table 2. Subgroup analysis of adjusted hazard ratio in 1-year outcomes for the comparison of ESRD and non-CKD groups

		Adverse outcomes			
Variable	reference	all-cause mortality	cardiovascular death	Hospitalization for CHF	[1]Hospitalization for ASCVD
ESRD	Non-CKD	2.77*** (2.05, 3.73)	1.87* (1.08, 3.24)	1.50* (1.04, 2.17)	2.33*** (1.69, 3.23)
Age (year)		1.04*** (1.03, 1.05)	1.03* (1.00, 1.05)	1.01 (1.00, 1.03)	1.01 (0.99, 1.02)
Male	Female	0.78 (0.59, 1.04)	1.50 (0.86, 2.61)	0.77 (0.53, 1.11)	1.01 (0.74, 1.37)
Comorbidity					
AKI	normal	1.51 (0.98, 2.33)	2.16 (0.99, 4.69)	2.34** (1.43, 3.81)	1.34 (0.79, 2.26)
Hypertension	normal	0.43*** (0.32, 0.59)	0.35** (0.20, 0.62)	0.86 (0.56, 1.32)	0.44*** (0.31, 0.62)
Hyperlipidemia	normal	0.47** (0.31, 0.72)	0.49 (0.21, 1.12)	0.76 (0.48, 1.20)	0.94 (0.65, 1.38)
AF	normal	1.22 (0.61, 2.44)	3.26* (1.29, 8.19)	1.47 (0.66, 3.28)	1.50 (0.72, 3.14)
COPD	normal	0.85 (0.59, 1.22)	0.61 (0.28, 1.32)	1.05 (0.68, 1.62)	0.92 (0.61, 1.38)
CLD	normal	1.73 (0.99, 3.02)	0.97 (0.29, 3.29)	0.57 (0.23, 1.42)	1.21 (0.67, 2.19)
Dementia	normal	1.21 (0.79, 1.87)	1.98 (0.92, 4.23)	1.58 (0.92, 2.73)	1.13 (0.65, 1.96)
Medications					
Diuretics	Not use	1.48* (1.02, 2.15)	1.80 (0.85, 3.83)	1.47 (0.91, 2.39)	0.90 (0.59, 1.38)
Antiplatelets	Not use	0.89 (0.61, 1.30)	1.05 (0.49, 2.23)	0.95 (0.59, 1.53)	0.70 (0.46, 1.07)
Warfarin	Not use	1.48 (0.71, 3.11)	1.97 (0.57, 6.77)	0.99 (0.31, 3.18)	0.90 (0.33, 2.50)
Rivaroxaban	Not use	0.58 (0.14, 2.45)		0.85 (0.11, 6.41)	1.28 (0.17, 9.57)
ACEIs/ARBs	Not use	0.92 (0.63, 1.33)	0.82 (0.38, 1.76)	0.59* (0.37, 0.96)	1.30 (0.85, 2.01)
Beta-2 blockers	Not use	1.55* (1.03, 2.34)	0.79 (0.35, 1.76)	1.26 (0.76, 2.10)	1.06 (0.67, 1.68)
CCBs	Not use	0.70 (0.48, 1.01)	0.89 (0.44, 1.83)	0.86 (0.54, 1.36)	0.76 (0.50, 1.17)
Statins	Not use	0.61* (0.38, 1.00)	0.68 (0.25, 1.86)	0.68 (0.37, 1.22)	1.00 (0.62, 1.60)
Metformin	Not use	1.22 (0.58, 2.58)	1.20 (0.26, 5.49)	1.58 (0.72, 3.45)	0.79 (0.35, 1.75)
Thiazolidinedione	Not use	0.75 (0.23, 2.41)	1.01 (0.13, 7.84)	1.27 (0.39, 4.16)	0.89 (0.27, 2.93)
Sulfonylureas	Not use	1.04 (0.61, 1.79)	0.76 (0.22, 2.62)	1.24 (0.65, 2.35)	1.24 (0.70, 2.20)
AGIs	Not use	0.94 (0.49, 1.79)	1.45 (0.41, 5.14)	1.00 (0.44, 2.26)	1.70 (0.87, 3.32)
DPP-4is	Not use	0.90 (0.58, 1.38)	0.78 (0.30, 1.99)	1.10 (0.64, 1.89)	1.24 (0.78, 1.97)
Insulin	Not use	1.67** (1.14, 2.44)	1.27 (0.60, 2.68)	1.53 (0.94, 2.49)	1.66* (1.09, 2.52)
GLP-1 RAs	Not use				

Cox proportional hazards regression, * $p < 0.05$ ** $p < 0.01$ *** $p < 0.0001$

[1]The study events for ASCVD:

(1) brain stroke (hemorrhagic stroke and ischemic stroke),

(2) acute myocardial infarction, and

(3) major adverse limb events (acute limb ischemia, major amputation, need for surgical or peripheral revascularization of peripheral artery occlusive disease).

1:1 matching on propensity score with index date, age, sex, comorbidities, and medications.

Abbreviations: ACEIs, angiotensin-converting enzyme inhibitors; AF, atrial fibrillation; AGIs, alpha-glucosidase inhibitors; ARB, angiotensin receptor blockers; AKI, acute kidney injury; ASCVD, atherosclerotic cardiovascular disease; CCBs, calcium channel blockers; CHF, congestive heart failure; CKD, chronic kidney disease; CLD, chronic liver disease; COPD, chronic obstructive pulmonary disease; DPP-4is, dipeptidyl peptidase 4 inhibitors; ESRD, end-stage renal disease; GLP-1 RAs, glucagon-like peptide 1 receptor agonists
